# Over-the-Counter Benzocaine Gel as a Cause of Methemoglobinemia: A Case Report

**DOI:** 10.7759/cureus.104949

**Published:** 2026-03-09

**Authors:** Anais Panossian, Audrey Sadra, Aram A Namavar

**Affiliations:** 1 Medicine, University of California, Los Angeles, USA

**Keywords:** acquired hemoglobinopathy, methemoglobinemia, oxidative drug reaction, refractory hypoxemia, topical anesthetic toxicity

## Abstract

Methemoglobinemia is a rare, potentially life-threatening disorder caused by oxidation of hemoglobin iron from the ferric (Fe^3+^) to the ferrous (Fe^2+^) state, impairing oxygen delivery to tissues. Although most cases of methemoglobinemia that are documented are reported after procedural use of benzocaine sprays, those occurring from over-the-counter benzocaine gel are uncommon and often underrecognized. We report a case of an elderly woman with multiple comorbidities who developed significant methemoglobinemia after prolonged Benzocaine use for oral discomfort. She presented with refractory hypoxemia unresponsive to escalating supplemental oxygen. Arterial blood gas analysis revealed elevated methemoglobin levels. Timely recognition and administration of methylene blue resulted in rapid resolution of symptoms. This case highlights the importance of considering methemoglobinemia in patients presenting with unexplained refractory hypoxia, especially in patients who report the use of local anesthetics.

## Introduction

Methemoglobinemia is an uncommon but potentially life-threatening hemoglobinopathy where the oxygen-carrying ability of hemoglobin is compromised due to its iron species being converted to the ferric (Fe^3+^) state from the normal ferrous (Fe^2+^) state [1-3]. This ultimately results in decreased oxygen delivery to tissues, leading to hypoxia. The disease can be congenital or acquired; the acquired form is more common and can occur from common agents, including but not limited to nitrites, sulfonamides, dapsone, chloroquine, and local anesthetics such as lidocaine and bupivacaine, that can cause either direct or indirect oxidation of hemoglobin [4,5]. Methemoglobinemia should be suspected in cases where dyspnea, cyanosis, or hypoxemia unresponsive to supplemental oxygen are present, particularly if there is known exposure to oxidative agents. Clinical manifestations can be variable and nonspecific, with severity being influenced by methemoglobin levels, rate of accumulation, clearance capacity, comorbidities, and extent of exposure. Symptoms can range from mild cyanosis and fatigue to severe hypoxia, altered mental status, and cardiovascular instability [3]. Treatment is often multi-fold and starts with removing the causative agent. If MetHb levels are <20%, supportive therapy with IV hydration and oxygen may suffice. However, methylene blue (1-2mg/kg administered intravenously over 5 minutes) is first-line for decreasing MetHb levels as it provides an electron to reduce Fe^3+^ back to the Fe^2+^ state [1]. If methylene blue is ineffective even after a second round of administration, other treatment options include ascorbic acid, exchange transfusion, or hyperbaric oxygen therapy [3].

## Case presentation

A 72-year-old female with a complex medical history, including recent diverticulectomy and cricopharyngeal myotomy, presented to the emergency department with progressive shortness of breath, chest tightness, and refractory hypoxia as measured by her home pulse oximeter. Following her recent ENT procedure, she observed persistently low oxygen saturation readings in the 80s despite supplemental oxygen use, prompting her to seek emergency care. The patient reported using an over-the-counter benzocaine-containing oral gel over the past year for oral discomfort related to dental retainers. She denied fever, chills, or cough, but noted mild cyanosis of her extremities and increasing dyspnea following her recent ENT procedure.

On presentation, she was alert and oriented, with mild respiratory distress. Despite escalation of supplemental oxygen therapy, including high-flow nasal cannula at 35 L/min with 88% FiO₂, her oxygen saturation remained suboptimal at 89%. Physical examination revealed mild cyanosis of the lips and nail beds, but no focal neurological deficits or cardiac murmurs. Chest examination showed mild subcutaneous crepitus over the lateral neck and upper chest, consistent with subcutaneous emphysema, but breath sounds were otherwise normal.

**Figure 1:**
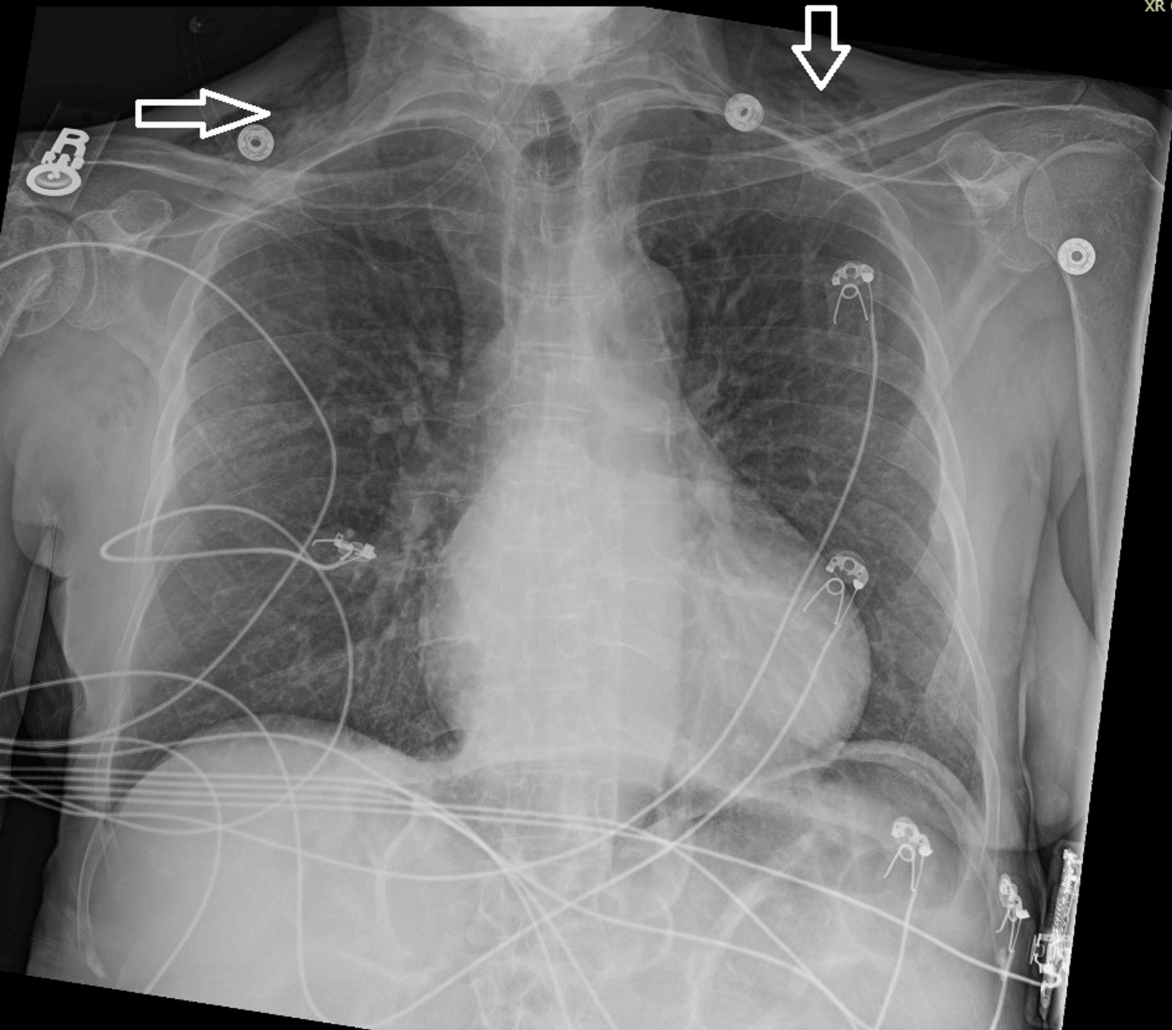
Chest X-ray Portable anteroposterior chest X-ray demonstrates extensive gas within the subcutaneous tissues of the upper chest wall bilaterally, consistent with subcutaneous emphysema (arrows). The lung parenchyma shows mild bronchial wall thickening without evidence of pulmonary edema, focal consolidation, pleural effusion, or pneumothorax. Cardiac silhouette is within normal limits. Chronic osseous deformities involving the left ribs and right humeral head/neck are again noted. External monitoring leads are present.

Laboratory evaluation demonstrated an arterial blood gas with co-oximetry showing a methemoglobin level of 14%, confirming the diagnosis of methemoglobinemia. Complete blood count and basic metabolic panel were within normal limits. Chest radiography demonstrated extensive subcutaneous emphysema involving the lower neck and upper chest wall, with no evidence of pneumonia, pulmonary edema, pleural effusion, or pneumothorax (Figure 1).

Upon review of her medication and exposure history, chronic benzocaine use, contained in the Orajel, was identified as the likely cause of her methemoglobinemia. Benzocaine can oxidize ferrous hemoglobin (HbFe²⁺) to ferric hemoglobin (MetHbFe³⁺), impairing oxygen binding and transport. Toxicology was consulted and recommended treatment with methylene blue, which acts as a cofactor for NADPH methemoglobin reductase, facilitating the reduction of MetHbFe³⁺ back to functional HbFe²⁺.

Given that supplemental oxygen cannot reverse the underlying hemoglobin oxidation, she was weaned off oxygen therapy. Following administration of methylene blue at a dose of 1.5 mg/kg over five minutes, her symptoms resolved rapidly within 30 minutes. As demonstrated in Table 1, the patient’s pre-treatment arterial sample demonstrated marked methemoglobinemia (14.7%) with elevated pO₂ (314 mmHg) and discordantly low oxygen saturation. Post-treatment venous co-oximetry showed normalization of methemoglobin to 1.9% and clinical resolution of hypoxia. She remained stable overnight and was discharged home the following day in good condition. The patient was advised to discontinue all benzocaine-containing products and follow up with her otolaryngologist for postoperative care.

**Table 1:** Arterial and Venous Blood Gas with Co-Oximetry Results Before and After Methylene Blue Administration. Arterial and venous blood gas with co-oximetry results shown before and after methylene blue administration. The patient’s pre-treatment arterial sample demonstrated marked methemoglobinemia (14.7%) with elevated pO₂ (314 mmHg) and discordantly low oxygen saturation. Post-treatment venous co-oximetry showed normalization of methemoglobin to 1.9% and clinical resolution of hypoxia. Note: Post-treatment results were obtained from a venous sample. Co-oximetry was performed on both arterial and venous specimens, demonstrating normalization of methemoglobin levels following therapy.

Parameter	Reference Range	Pre-Treatment, (Arterial)	Post-Treatment, (Venous)
pH	7.35–7.45	7.51	7.41
pCO₂ (mmHg)	35–45	37	51
pO₂ (mmHg)	80–100 (arterial)	314	37
Bicarbonate (mmol/L)	22–26	29.3	32.2
Base Excess (mmol/L)	≤ 3	6	8
O₂ Saturation (%)	≥ 94 (arterial)	97	64.6
Hemoglobin (g/dL)	12–16	10.2	10.1
Oxyhemoglobin (%)	≥ 94	83.1	53.1
Carboxyhemoglobin (%)	≤ 1.5	<1.0	<1.0
Methemoglobin (%)	≤ 1.5	14.7	1.9

## Discussion

Methemoglobinemia is an uncommon but potentially life-threatening condition in which hemoglobin is oxidized to the ferric (Fe^3+^) state, impairing oxygen delivery and resulting in tissue hypoxia. Although this condition can be congenital or acquired, the acquired form, typically triggered by oxidising medications, is far more common. Methemoglobinemia should be considered in patients with dyspnea, cyanosis, or hypoxemia refractory to supplemental oxygen, especially in the setting of known or suspected exposure. While the general mechanisms and clinical features of methemoglobinemia are well described in the literature, our case highlights an atypical context of exposure and several diagnostic challenges that distinguish it from more frequently reported cases. 

Most published cases of methemoglobinemia occur after high-dose, procedural exposure to benzocaine sprays, particularly during endoscopy, bronchoscopy, or dental procedures [4,6,7]. In these settings, methemoglobin levels frequently exceed 20%, with abrupt symptom onset occurring within minutes [4,7,8]. A review of 242 published cases revealed benzocaine as responsible for 66% of topical anesthetic-related methemoglobinemia events, with sprays disproportionately represented compared to gels or creams [9].

In contrast, our case presents an instance of chronic, low-dose outpatient exposure to an over-the-counter gel. This presentation is uncommon, with only a limited number of cases being reported [5,6], underscoring the challenge in recognition, particularly in the ambulatory setting. Moreover, the benzocaine gel concentration is much lower than the highly concentrated sprays implicated in most cases, which likely explains the patient’s more moderate methemoglobin level of 14% at presentation, still clinically meaningful but below levels typically associated with severe toxicity. The patient’s rapid symptomatic and biochemical response to 1.5 mg/kg of methylene blue mirrors findings from multiple published reports demonstrating prompt reversal of methemoglobinemia with first-line therapy and highlights the effectiveness of targeted therapy when the diagnosis is made [4].

Several factors in our case aligned with known risk factors described in prior case reports, including advanced age, recent mucosal disruption, and underlying comorbidities [4,5,7,10]. Reports have also shown that recent mucosal injury can significantly increase systemic absorption of topical anesthetics [8], and our patient’s recent ENT surgery likely contributed to heightened vulnerability. This, in combination with her pattern of chronic use, daily application over months, may also have allowed for cumulative oxidative stress. Her presentation with refractory hypoxia despite high-flow supplemental oxygen is characteristic of methemoglobinemia, yet can initially mimic postoperative complications such as pulmonary embolism, aspiration, or airway injury. These intersecting features increased diagnostic complexity and underscore the importance of a high index of suspicion, particularly when pulse oximetry readings remain fixed in the 80s despite escalating oxygen therapy.

Overall, this case contributes to the limited body of literature on chronic outpatient benzocaine exposure as a cause of methemoglobinemia. It also underscores the importance of recognizing non-prescription medications as potential sources of toxicity, especially in older adults who may self-medicate for chronic discomfort. Given the widespread availability of over-the-counter benzocaine gels, greater clinician and patient awareness is needed to prevent delayed diagnosis and unnecessary diagnostic or therapeutic interventions.

## Conclusions

This case illustrates a rare but serious complication of a widely available over-the-counter product, highlighting the importance of clinical vigilance in recognizing atypical causes of hypoxia. Chronic benzocaine exposure, even at low doses, can lead to significant methemoglobinemia, particularly in elderly individuals or those with comorbidities such as cardiopulmonary disease. Early identification and prompt administration of methylene blue can lead to rapid clinical improvement and full recovery. Clinicians should maintain a high index of suspicion for methemoglobinemia in patients with unexplained cyanosis or refractory hypoxemia, especially when pulse oximetry readings are discordant with clinical appearance. Methemoglobinemia remains underreported in the outpatient setting, and further studies are needed to understand optimal strategies for diagnosis, treatment, and prevention. Additionally, this case also underscores the importance of patient education on the safe use of topical anesthetics and for regulatory attention to labelling and warnings on nonprescription benzocaine-containing products.
